# Supplementary material for: A Novel Endo-Polygalacturonase from Penicillium rolfsii with Prebiotics Production Potential: Cloning, Characterization and Application
Source: Foods. 2022 Nov 1;11(21):3469. doi: 10.3390/foods11213469 (PMC9656037; doi:10.3390/foods11213469)
Supplement: Supplementary file 1 [file foods-11-03469-s001.zip › foods-1943433-supplementary.pdf]

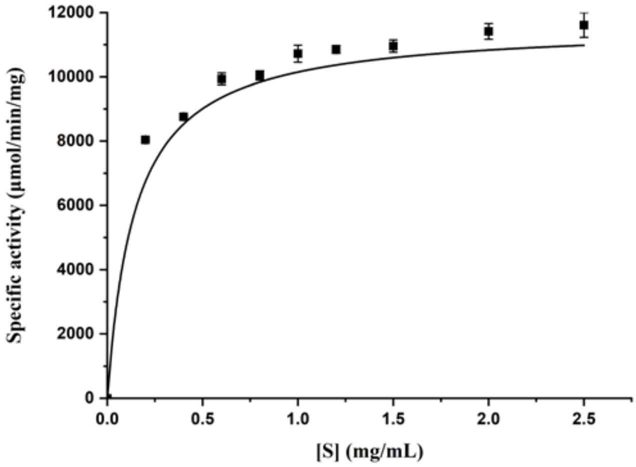

Figure S1. The effect of substrate concentration on enzyme activity. The enzyme activity was measured with 0.4-2.5 mg/mL polygalacturonan acid as the substrate under 60 °C at 50 mM pH 6.0 Na_2HPO_4-citric acid buffer for 3 min.

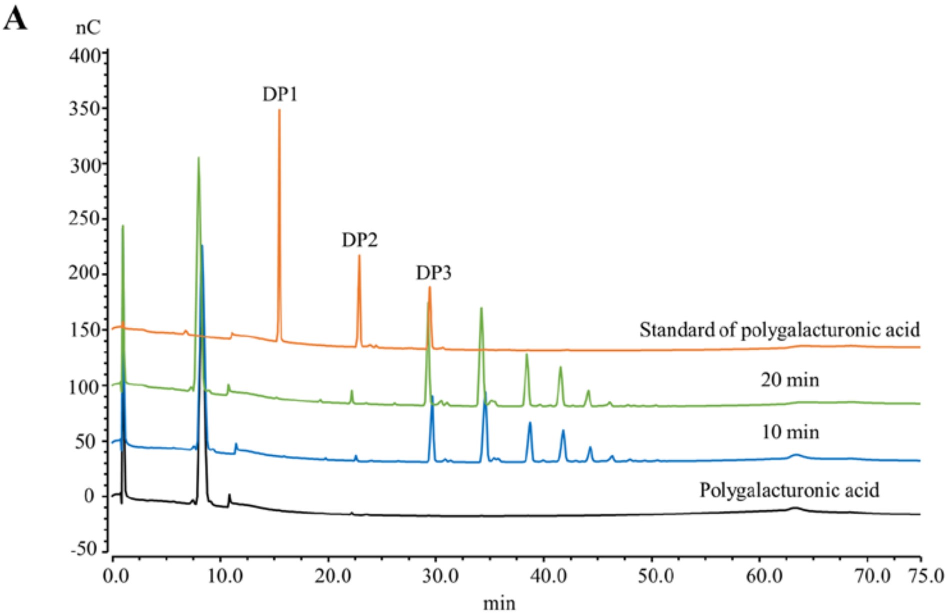

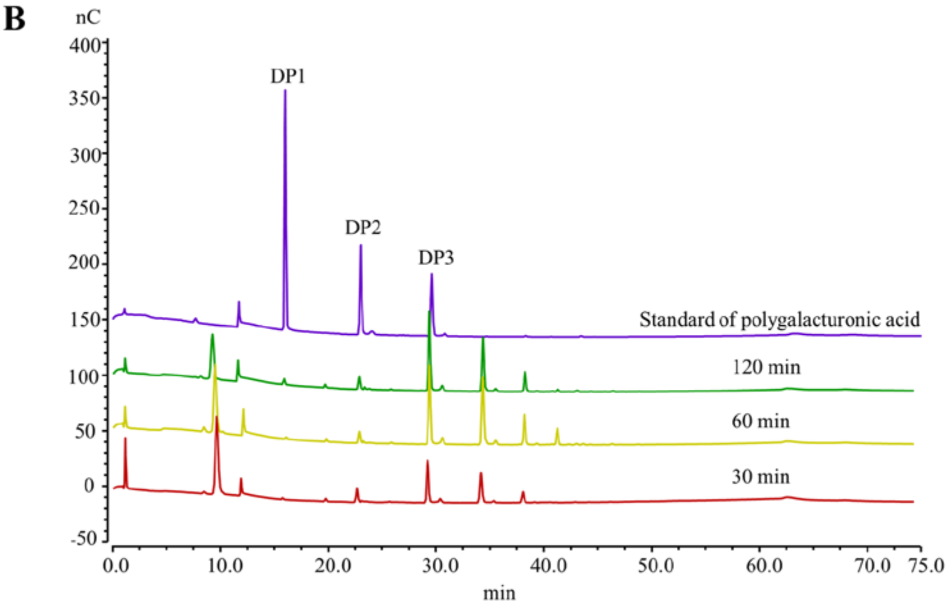

Figure S2. The chromatograms of HPAEC-PAD analysis for hydrolysis products by pePGA on polygalacturonic acid. (**A**) Hydrolysis products from 0 to 20 min; (**B**) Hydrolysis products from 30 min (5-fold dilution for detection), 60 min (5-fold dilution for detection) and 120 min (10-fold dilution for detection). DP is the average degree of polymerization at peak.

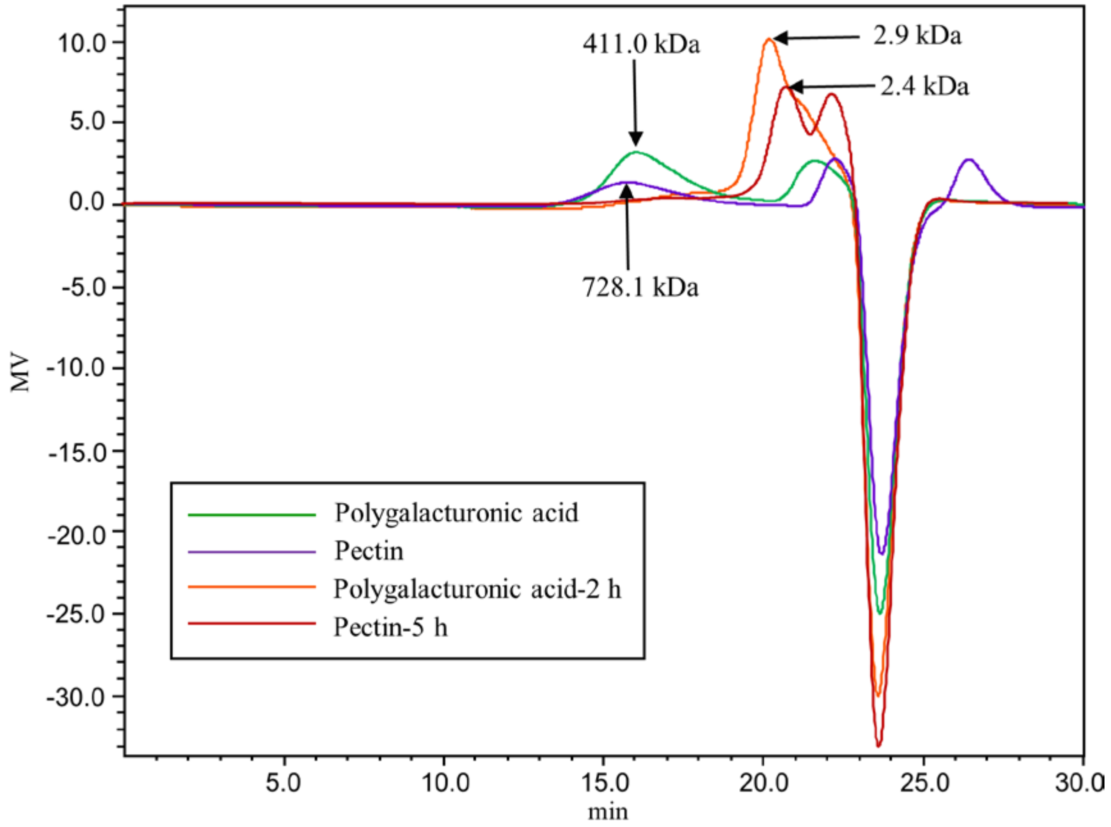

Figure S3 The high-performance gel filtration chromatography analysis for hydrolysis products by pePGA on polygalacturonic acid and commercial demethylated pectin.

In order to reveal the effect of mono-galacturonic acid in pePGA-POS on antimicrobial activity, TLC analysis was used to determine the mono-galacturonic acid content in pePGA-POS. The results are illustrated in Figure S4, the mono-galacturonic acid accounted for about 10% of pePGA-POS. The antimicrobial activity of pePGA-POS was determined by using a 400 mg/mL pePGA-POS solution，so we determined that the mono-galacturonic acid concentration used for the antimicrobial activity experiment was 40 mg/mL.

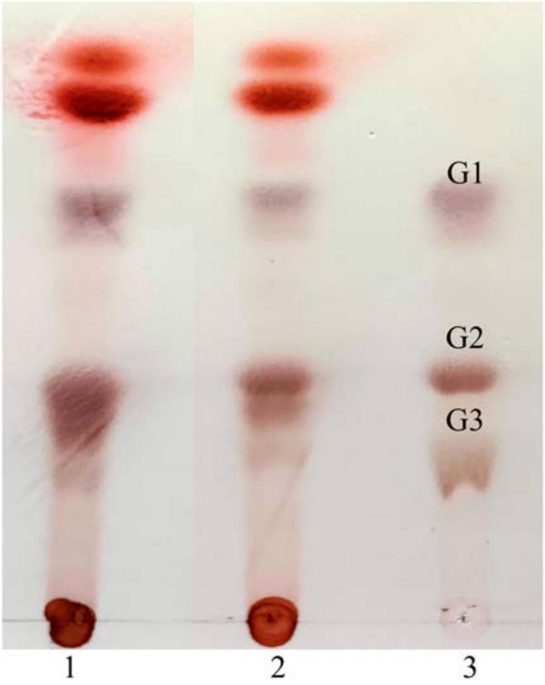

Figure S4. TLC analysis of pePGA-POS at different concentrations. lane 1, 20 mg/mL pePGA-POS; lane 2, 10 mg/mL pePGA-POS; Lane 3, mixture of mono-galacturonic acid (G1, 1 mg/mL), di-galacturonic acid (G2, 1 mg/mL) and tri-galacturonic acid (G3, 1 mg/mL).

During our research, we found that pH has a great influence on the antimicrobial activity of pePGA-POS, so we tested the antimicrobial activities of pePGA-POS and mono-galacturonic acid at different pH. Since the initial pH of mono-galacturonic acid dissolved in water (40 mg/mL) is 2.0, which is lower than the initial pH (3.0-3.5) of 400 mg/mL pePGA-POS dissolving in water, we adjusted the pH of mono-galacturonic acid solution (40 mg/mL) to 3.0-3.5 with 0.5 M NaOH and tested its antimicrobial activity. In addition, we also tested the antimicrobial activities of 40 mg/mL mono-galacturonic acid and 400 mg/mL pePGA-POS at pH 5.0, which were adjusted the pH with 0.5 M NaOH.

As shown in Figure S5, the pePGA-POS solution and mono-galacturonic acid solution had antibacterial activities against *B. subtilis* 168, *S. aureus* and *E. coli* JM109 at their initial pH, and the pePGA-POS solution showed higher antimicrobial activity. However, the antimicrobial activity of mono-galacturonic acid was lost when the pH was adjusted to 3.0-3.5. This indicated that oligomeric galacturonic acid in pePGA-POS at its intial pH (3.0-3.5) rather than mono-galacturonic acid played an antimicrobial role in pePGA-POS. We further adjusted the pH of pePGA-POS solution and mono-galacturonic acid solution to 5.0, and we found that both lost their antimicrobial activities. We speculated that the antimicrobial activity of pePGA was related to the pH of the

oligomeric galacturonic acid or mono-galacturonic acid with different protonation state.

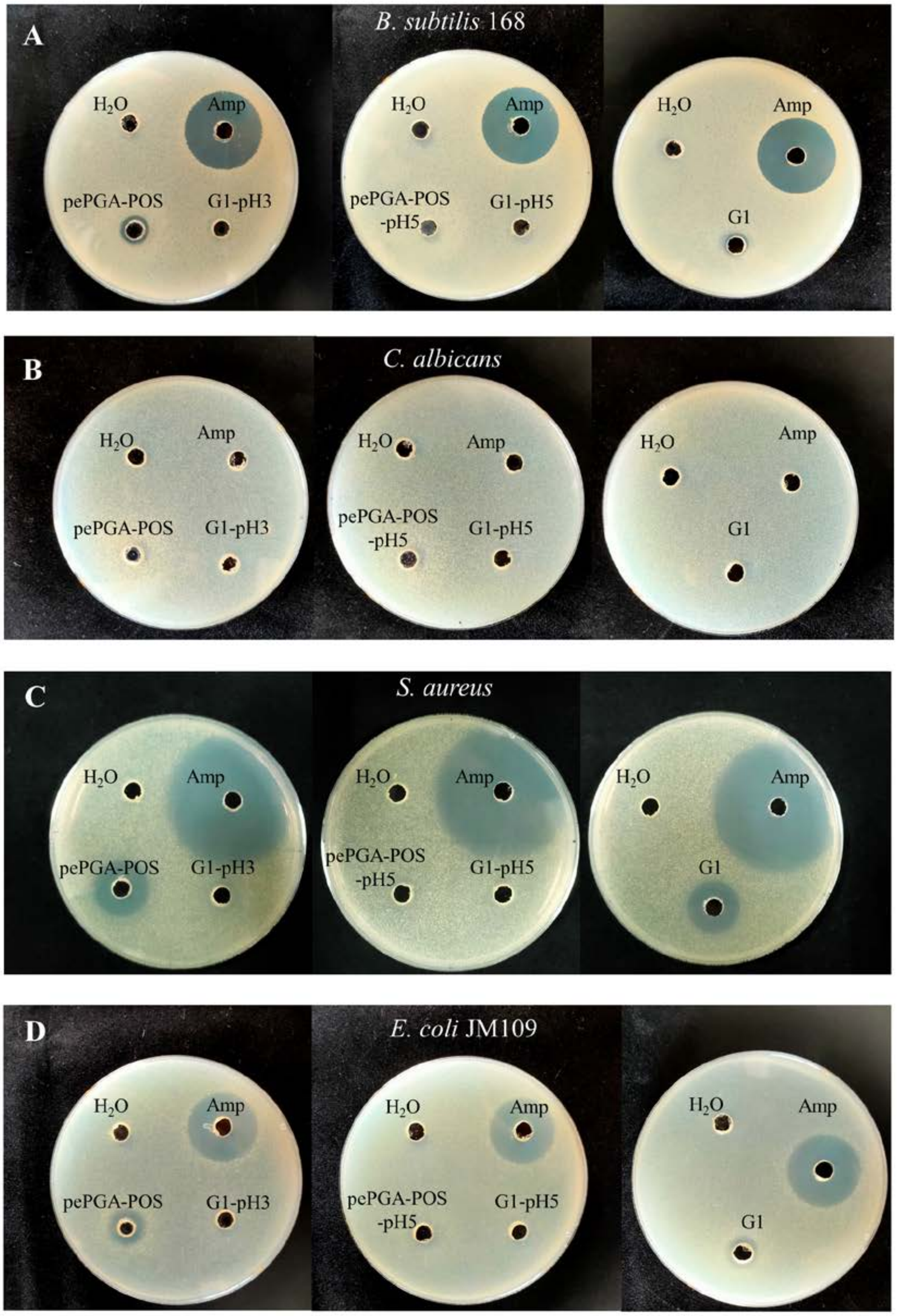

Figure S5. Antibacterial activities of pePGA-POS and mono-galacturonic acid against (A) *B. subtilis* 168; (B) *C. albicans*; (C) *S. aureus*; (D) *E. coli* JM109. PePGA-POS: 400 mg/mL pePGA-POS solution with pH 3.0-3.5; G1-pH 3: 40 mg/mL mono-galacturonic acid solution with pH 3.0-

3.5; pePGA-POS-pH 5: 400 mg/mL pePGA-POS solution with pH 5.0; G1-pH 5: 40 mg/mL mono-galacturonic acid solution with pH 5.0; G1: 40 mg/mL mono-galacturonic acid solution with initial pH 2.0.

As shown in Figure S6, the antimicrobial activity of mono-galacturonic acid increased with increasing concentration, however, the antimicrobial activity of mono-galacturonic acid was lost when the pH was adjusted to 5.0, although at a concentration of 200 mg/mL.

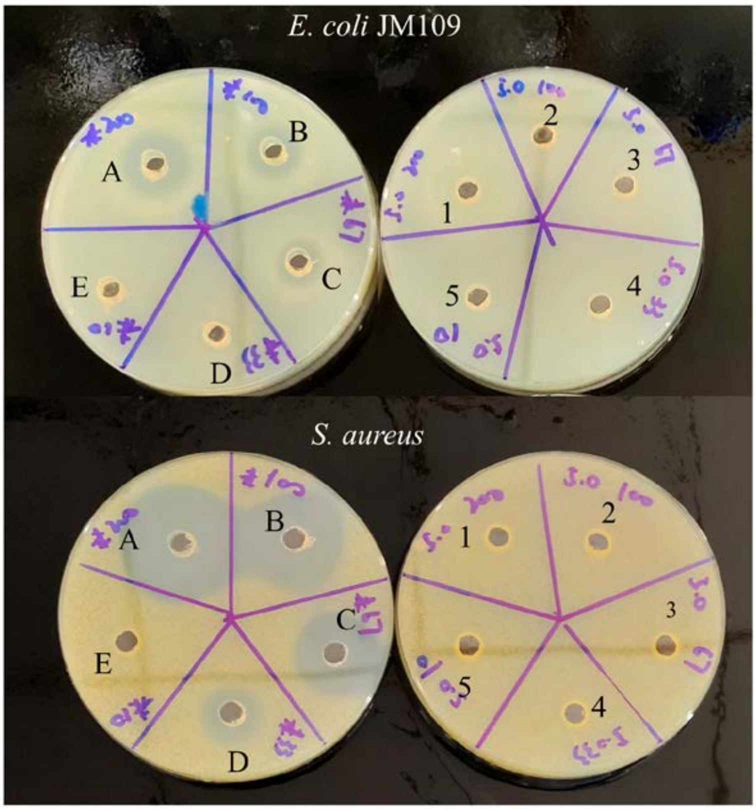

Figure S6. Antibacterial activities of mono-galacturonic acid against *E. coli* JM109 and *S. aureus*. A: 200 mg/mL mono-galacturonic acid with pH 1.5-2.0; B: 100 mg/mL mono-galacturonic acid with pH 2.0; C: 67 mg/mL mono-galacturonic acid with pH 2.0; D: 33 mg/mL mono-galacturonic acid with pH 2.0; E: 10 mg/mL mono-galacturonic acid with pH 2.0-2.5; 1: 200 mg/mL mono-galacturonic acid with pH 5.0; 2: 100 mg/mL mono-galacturonic acid with pH5.0; 3: 67 mg/mL mono-galacturonic acid with pH 5.0; 4: 33 mg/mL mono-galacturonic acid with pH 5.0; 5: 10 mg/mL mono-galacturonic acid with pH 5.0;

In addition to the above, we also tested the antimicrobial activities of the enzymatically degradation products from citrus pectin (POS-citrus), the enzymatically degradation products from apple pectin (POS-apple), mannuronic acid oligosaccharides (MOS) and carrageenan oligosaccharides (COS). The citrus pectin and apple pectin were purchased from Hongxing (Henan, China) with 60-70% degree of esterification. The MOS and COS were purchased from Qingdao HEHAI Biotech Co., Ltd (Shandong, China).

One hundred milliliters of pectin (4% w/v), 4000 U pePGA and 40 mg pectin methyl esterase were incubated at 40 °C for 5 h, respectively. The mixture was boiled for 20 min in water to inactivate enzymes. Then, POS-citrus and POS-apple were obtained by centrifuging the mixture at 6,300 × g

for 20 min and freeze-drying of the supernatant.

The 200 mg/mL POS-citrus solution and 200 mg/mL POS-apple solution were used to determine the antimicrobial activities. Both solutions had pH values of 2.5 and also were adjusted the pH to 5.0 with 0.5 M NaOH to determine the antimicrobial activities. In order to prepare a high concentration solution of MOS and COS as much as possible, we first prepared MOS and COS into supersaturated solutions, heat them in a boiling water bath for 8min to accelerate the dissolution of the substance, and then the insoluble substance was removed to obtain high concentration solutions of MOS and COS (concentration less than 400 mg/mL). The pH of MOS solution was 4.8-5.1. The pH of COS solution was 3.5 and the COS solution also was adjusted the pH to 5.0 with 0.5 M NaOH to determine the antimicrobial activities.

As shown in Figure S7, the POS-citrus and POS-apple showed good antimicrobial activities against *B. subtilis* 168, *S. aureus* and *E. coli* JM109 at their initial pH, and the antimicrobial activities lost when the pH was adjusted to 5.0. A previous study [36] has demonstrated that a kind of mannuronic acid fraction, M3 (molecular weight 4.235 kDa) had high inhibitory activity against *E. coli*, *S. aureus* and *B. subtilis*, and Guo *et al*. [37] reported that carrageenan oligosaccharides possessed excellent antibacterial activity. However, the MOS and COS had no antimicrobial activity in this study.

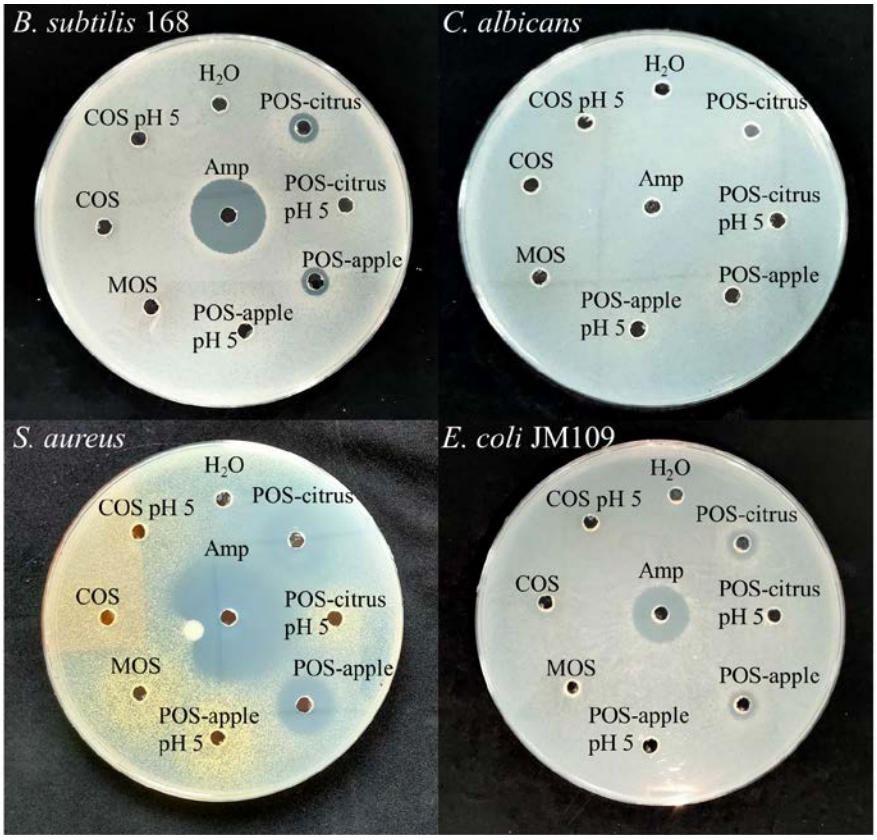

Figure S7. Antibacterial activities of POS from citrus pectin (POS-citrus), POS from apple pectin (POS-apple), mannuronic acid oligosaccharides (MOS) and carrageenan oligosaccharides (COS) against *B. subtilis* 168, *C. albicans*, *S. aureus* and *E. coli* JM109.
